# “Could a subset of joint mobility tests define generalized joint hypermobility?”: A descriptive observational inception study

**DOI:** 10.1371/journal.pone.0298649

**Published:** 2024-04-18

**Authors:** Angela Schlager, Lena Nilsson-Wikmar, Kerstin Ahlqvist, Christina B. Olsson, Per Kristiansson

**Affiliations:** 1 Department of Public Health and Caring Sciences, Uppsala University, Uppsala, Sweden; 2 Department of Neurobiology, Care Sciences and Society, Division of Physiotherapy, Karolinska Institutet, Huddinge, Sweden; 3 Academic Primary Healthcare Centre, Region Stockholm, Stockholm, Sweden; Aichi Prefectural Mikawa Aoitori Medical and Rehabilitation Center for Developmental Disabilities, JAPAN

## Abstract

**Background:**

Generalized joint hypermobility is an inherited collagen phenotype based on clinical assessments of joint mobility. However, there is no international consensus to define generalized joint hypermobility, both considering which joint mobility tests should be included and limits for joint hypermobility.

**Objectives:**

The primary aim of the study was to identify a subset of joint mobility tests to define generalized joint hypermobility. A further aim was to evaluate standardized limits for the classification of hypermobility in different joint types throughout the body.

**Methods:**

A total of 255 early pregnant women were included in the study. Joint mobility was measured according to a structured protocol. Correlation and principal component analysis were used to find a subset of joint mobility tests. To classify hypermobility in each joint mobility test, five different standard deviation levels plus 0.84, plus 1.04, plus 1.28, plus 1.64 and plus 2 were used, corresponding to 20%, 15%, 10%, 5% and 2.5% of the normal distribution.

**Results:**

No subset of joint mobility test could define generalized joint hypermobility. The higher the standard deviation levels, the higher the limit to classify joint hypermobility and the lower the prevalence. As a result of no subset of joint mobility tests were found to define generalized joint hypermobility, different combinations of major and minor joints in upper and lower limbs and the axial skeleton, were systematically developed. These combinations were evaluated for each standard deviation level, resulting in a prevalence of generalized joint hypermobility between 0% and 12.9% and a clear variation in how the hypermobile joint mobility tests were distributed.

**Conclusion:**

It is probably not possible to choose a subset of joint mobility tests to define GJH. In order not to overlook generalized joint hypermobility, a broader assessment of different joint types and sizes of joints appears to be needed. The prevalence of generalized joint hypermobility is dependent on joint hypermobility limit and the chosen combination of joint mobility tests.

## Introduction

Generalized joint hypermobility (GJH) is an inherited collagen phenotype [1]. To date, the genetic basis of GJH is unknown and GJH is based on clinical assessments on joint mobility [2]. However, there is no international consensus to define GJH [3–6]. Since GJH is the main criterion for heritable connective tissue disorders, like Ehlers-Danlos Syndromes [7] the definition of GJH is crucial.

Over previous years, several scores to define GJH have been developed with different sets of joint mobility tests and cut-off levels. Carter & Wilkinson score consists of five unilateral joint mobility tests, apposition of the thumb to the forearm, dorsifexion of the ankle, as well as hyperextension of the elbows, knees and all the metacarpophalangeal joints [8]. The Beighton score (BeS) was modified from the Carter & Wilkinson with bilateral assessment with hyperextension of all the metacarpophalangeal joints, replaced by hyperextension of the metacarpophalangeal joint in the fifth finger and dorsifexion of the ankle, replaced by the palms to floor test [9]. The Contompasis score is a further modification of the BeS with an additional test of calcaneus valgus mobility and by grading the joint mobility [10]. In addition, the Hospital del Mar criteria (HdM) offers eight joint mobility tests. Except for apposition of the thumb to the forearm and hyperextension of the fifth finger, additional tests for shoulder, ankle, knee, patella, hip and big toe are assessed, all on the non-dominant bodyside [11]. Two more recent assessment scores for GJH, divided into Upper and Lower Limb Assessment Scores, contain bilateral joint mobility tests and tests for instability and translation of the shoulder, elbow and wrist as well as the hip, knee, ankle and foot respectively, with a unilateral final score [12, 13]. All of the mentioned scores consist of dichotomous assessments of joint mobility with varied limits for the classification of hypermobility in each joint mobility test and cut-off level to define GJH. The clinimetric properties of these GJH scores are weak or not evaluated [11–15]. This also applies to the Beighton score despite its common use as base score for GJH in heritable connective tissue disorders [7]. Limits for the classification of hypermobility in each joint mobility test are not well studied and not clearly assigned [3, 16]. Plus 2 standard deviation (SD) from the mean of normal distribution of joint mobility is recommended [17]. Plus 2 SD is a general, consensus-based estimate, used in other disciplines [16]. Finally, it is unclear how to perform the joint mobility assessment and in most scores no regard is taken to correlated bilateral joint tests [4, 16–19].

A proposed definition for GJH includes the presence of joint hypermobility simultaneously at upper and lower limbs and the axial skeleton with involvement of major and minor joints [4, 5], already mentioned in 1964 by Carter & Wilkinson [8]. Thus, to the best of our knowledge, there is no evidence-based evaluation of this proposed definition for how and which joint mobility tests should be included, or limits for joint hypermobility that accurately reflect GJH.

The primary aim of the study was to identify a subset of joint mobility tests to define generalized joint hypermobility. A further aim was to evaluate standardized limits for the classification of hypermobility in different joint types throughout the body.

## Materials and methods

### Study design

This is a descriptive observational study including 255 women, consecutively recruited in early pregnancy, from two maternal health care centres, in a medium-sized city in Sweden. The maternal health care centres were selected by convenience. The study is part of a prospective inception cohort study with an overall aim to investigate generalized joint hypermobility, by different definitions, and its relation to pregnancy related pain.

### Study population

In Sweden, pregnant women have maternity care free of charge. That includes regular maternal health care visits throughout the pregnancy. During the study inclusion period from 15^th^ of february 2014 to 15^th^ of February 2019, approximately 5,500 women visited the two maternal health care centres. Midwives were instructed to invite all who met the inclusion criteria. A letter of information about the study was handed out or sent home. Women who wanted to, contacted the study supervisors for additional information. The inclusion criteria were an estimated duration of gestation less than 16 completed weeks and to read the Swedish language. There were no exclusion criteria, however joints with inflammation, spasticity, joint replacement or musculoskeletal injury were excluded from the assessment of joint mobility. A total of 255 pregnant women were included in the study.

### Data collection

Socio-demographical data was completed using a web-based questionnaire in privacy before the clinical examination. The questionnaire included questions about previous children, origin, highest completed education, marital status, smoking one month before pregnancy and right or left handed.

Anthropometric data: height (cm) and weight (kg) were clinically assessed. Height was measured without shoes with a wall-mounted tape measure to the nearest centimeter. Weight was measured with indoor dressing on a balance lever. Body mass index (BMI) was calculated as kg/m^2^.

The joint mobility tests were chosen in order to contain anatomical differences such as joint type and articular soft tissue structure, major and minor joints in upper and lower limbs and the axial skeleton. The joint mobility tests encompassed bilateral tests of shoulder external rotation, elbow extension, fifth finger extension, thumb apposition, knee extension and calcaneus valgus test. Unilateral measurement on the non-dominant body side of hip abduction, hip external and internal rotation, patella medial-lateral, foot flexion and big toe extension and the palms to floor test were performed. An ordinal scale was used for thumb apposition, patella medial-lateral and palms to floor test and a continuous angle measurement to the nearest degree was used for the other joint mobility tests. The reliability for the joint mobility tests were moderate to high [20].

In total 255 participants were measured in the shoulder external rotation, fifth finger extension, elbow extension, palms to floor, knee extension and calcaneus valgus test. In total 243 were measured in hip external-and internal rotation, big toe extension and foot flexion and 242 participants were measured in patella medial-lateral and hip abduction. The difference in the number of participants measured, were due to the assessors starting some joint mobility tests after study start. Totally 240 women were measured in all 13 joint mobility tests.

According to a structured protocol, two physiotherapists completed the joint mobility tests. The structured protocol comprised written information about start position of body part, position of goniometer, anatomic landmarks, stabilisation of adjacent structures and using active or passive mobility, illustrated with photos [20]. To standardise performance and increase inter-tester reliability, the two assessors compared and aligned their test results in pre-study training sessions.

### Statistical analysis

Summary statistics were calculated using standard methods. As a first step to find a subset of joint mobility tests to define GJH, the Spearman’s correlation coefficient ρ (rho) was used to explore correlations between the included joint mobility tests. In the next step, the principal component analysis (PCA), with orthogonal varimax rotation, was conducted to find a subset of joint mobility tests that accurately describes the variation of all joint mobility tests to define GJH [21, 22]. In order to the joint mobility tests would be comparable in the analyses, a z-score was computed. The extraction of components was based on a minimum eigenvalue at >1.0, the Kaiser criterion, and component loading of at least 0.35 after rotation [21, 22]. To reach the best fit to the data, the analysis was checked for crossloadings, freestanding items and components with few items [21, 22]. Furthermore, to measure sampling adequacy, the Kaiser-Meyer-Olkin Measure of Adequacy (KMO), with a threshold of >0.60 was used [23]. For the assessment of internal consistency reliability, the Cronbach alpha (α) value was set to α >0.7 [24].

To evaluate different limits to classify hypermobility of each joint mobility test, five different standard deviation (SD) levels, the uppermost of a normal distribution were used, plus 0.84 SD, plus 1.04 SD, plus 1.28 SD, plus 1.64 SD and plus 2 SD. This corresponds to the uppermost 20%, 15%, 10%, 5% and 2.5% of the normal distribution.

All analyses were carried out using Statistical analysis system, version 9.4.

### Ethics

Informed written consent was provided from all participants. Before signing the consent form, the women were informed that participation was voluntary and what to expect from the clinical examinations. The study was approved by the Regional Ethical Review Board in Uppsala, Sweden, reference number 2013/186. The study complied with the principles of the Declaration of Helsinki.

## Results

Table 1 shows the characteristics of the 255 women with an average of 12 completed weeks of gestation. The majority had European origin, were right-handed, had completed university education, had a partner, were non-smokers and were first-time mothers.

**Table 1:** Baseline characteristics of 255 women in early pregnancy. Data presented as mean or number.

Variable	n	
Age (years)^a^	254	32 (4.4)
Height (m)^a^	252	1.67 (0.06)
Weight (kg)^a^	254	67 (11.8)
Body mass index (kg/m^2^)^a^	252	24 (3.8)
Completed weeks of gestation^b^	255	12 (2.1)
Nulliparous, n (%)	254	151 (59.4)
Origin in Europe, n (%)^c^	254	237 (93.3)
Completed university education, n (%)	254	208 (81.9)
Having a partner, n (%)	253	241 (95.3)
Non-smoker, n (%)^d^	252	245 (97.2)
Right-handed, n (%)	255	237 (92.9)

^a^Mean and standardeviations

^b^Estimated by ultrasound or in case of missing ultrasound date, with last menstrual period

^c^The participant and both parents born in Europe

^d^One month before pregnancy

Table 2 displays the distribution of joint mobility in the 19 joint mobility tests among the 255 women, representing different joint types, major and minor joints in upper and lower limbs and the axial skeletion. The different joint mobility tests showed a wide range and the bilateral tests showed a similar joint mobility.

**Table 2:** Distribution of 19 joint mobility tests results among 255 women in early pregnancy.

Joint mobility measurement		Joint mobility	
	n	Mean (SD)	Range
Shoulder external rotation left^a^	255	64 (16.5)	20 to100
Shoulder external rotation right^a^	255	68 (14.9)	30 to 102
Elbow extension left^b^	255	6 (4.7)	0 to -20
Elbow extension right^b^	255	6 (4.6)	0 to -18
Fifth finger extension left^c^	255	77 (12.4)	45 to 115
Fifth finger extension right^c^	255	73 (13.9)	30 to 112
Thumb apposition left^d^	254	1 (0.7)	1 to 4
Thumb apposition right^d^	255	1 (0.7)	1 to 4
Palms to floor^e^	255	2 (1.3)	1 to 5
Hip abduction^f^	242	35 (6.7)	19 to 53
Hip external rotation^g^	243	44 (9.6)	10 to 78
Hip internal rotation^h^	243	51 (10.1)	30 to 82
Knee extension left^i^	255	4 (3.9)	0 to -17
Knee extension right^i^	255	4 (3.9)	0 to -19
Patella medial-lateral^j^	242	3 (0.9)	2 to 6
Foot flexion^k^	243	36 (7.1)	21 to 61
Calcaneus valgus left^l^	255	3 (1.9)	0 to 8
Calcaneus valgus right^l^	255	2 (2.0)	0 to -10
Big toe extension^m^	243	98 (12.6)	57 to 125

A goniometer was used in joint mobility tests and measured to the nearest one degree, except for thumb apposition, palms to floor and patella medial-lateral, which were ordinal variables.

^a^ Shoulder external rotation was assessed by active joint mobility measured in degrees with the participant seated with the upper arms adducted close to the body with elbows flexed to 90 degrees, with the forearm in mid position and thumbs facing up.

^b^ Elbow extension was assessed by passive joint mobility measured in degrees with the participant seated, the arm along the body and forearm in full supination.

^c^ Fifth finger extension was assessed by passive range of motion measured in degrees with the participant seated with the forearm resting on a table with the palm down, the elbow flexed and the adjacent finger stabilized.

^d^Apposition of the thumb was assessed by passive joint mobility with the participant seated, leaning the elbow on a table and was divided into 4 scores, where 1 = no contact with the forearm, 2 = thumb touches forearm, 3 = thumb digs into forearm easily, 4 = thumb overlaps outside of forearm. Ordinal variable.

^e^ Palms to floor was assessed with the participant standing through active flexion of the trunk with the knees extended. Palms to floor was divided into 6 scores, where 1 = no contact with the floor, 2 = fingertips touching floor, 3 = fingers touching floor, 4 = palms to floor, 5 = wrists to floor, 6 = forearms to floor. Ordinal variable.

^f^ Hip abduction was assessed by passive joint mobility measured in degrees with the participant in supine position with straight knees and toes pointing toward the ceiling. No movement was allowed in the pelvis.

^g^ Hip external rotation was assessed by passive joint mobility measured in degrees with the participant in sitting with the hip to be measured in 90 degrees flexion and neutral in rotation. The knee is flexed 90 degrees with a towel placed under the distal part of the thigh to maintain the horizontal position of the thigh. The leg not to be measured was abducted in the hip joint and the foot is placed on a stool. The subject stabilized with the hands.

^h^ Hip internal rotation was assessed as the hip external rotation.

^i^ Knee extension was assessed by passive joint mobility measured in degrees with the participant in supine position with knees straight. The heel was placed on a 10 cm high block under with the knee hanging freely in the air.

^j^ Patella medial-lateral was assessed with the participant in supine with straight knees. Patella was divided into 4 longitudinal quadrants and was passively brought medial and lateral with a total range between 0–8 quadrants. Ordinal variable.

^k^ Foot flexion was assessed by active joint mobility measured in degrees with the participant half kneeling with hip and knee in 90 degrees, the foot to be measured in front, fully stabilized against the floor.

^l^ Calcaneus valgus test was assessed with the participant standing, groin width distance between feet.

^m^ Big toe extension was assessed by passive joint mobility measured in degrees with the participant in supine position with ankles and toes in neutral position.

Table 3 shows a matrix of the correlations between the 19 joint mobility tests. Bilateral symmetric joint mobility tests showed the highest correlation coefficients, 0.59 to 0.88. Some body close joint mobility tests, fifth finger extension and thumb apposition left and right, patella medial-lateral and knee extension right, showed correlation coefficients, 0.40 to 0.46. The other joint mobility tests showed correlation coefficients, -0.10 to 0.38. To avoid bias from the high correlations between the bilateral symmetric joint mobility tests, 13 joint mobility tests on the non-dominant body side, are used in the further analyses. These joint mobility tests were shoulder external rotation, elbow extension, fifth finger extension, thumb apposition, palms to floor test, hip abduction, hip external, internal rotation, knee extension, patella medial-lateral, foot flexion, calcaneus valgus test and big toe extension.

**Table 3:** Spearman’s correlation coefficients between 19 joint mobility tests results among 255 women in early pregnancy.

	**1**	**2**	**3**	**4**	**5**	**6**	**7**	**8**	**9**	**10**	**11**	**12**	**13**	**14**	**15**	**16**	**17**	**18**	**19**	
**1**	**1.00**																			
**2**	***0*.*85***	**1.00**																		
**3**	0.36	0.31	**1.00**																	** 1.00**
**4**	0.32	0.26	***0*.*88***	**1.00**																
**5**	0.08	0.08	0.22	0.20	**1.00**															
**6**	0.14	0.14	0.26	0.24	***0*.*78***	**1.00**														
**7**	0.20	0.19	0.21	0.18	0.42	0.45	**1.00**													***0*.*59–0*.*88***
**8**	0.24	0.24	0.24	0.21	0.40	0.46	***0*.*88***	**1.00**												
**9**	0.10	0.14	0.16	0.10	0.19	0.15	0.07	0.07	**1.00**											
**10**	0.25	0.26	0.03	-0.01	0.22	0.16	0.22	0.26	0.27	**1.00**										
**11**	0.05	0.12	0.14	0.14	0.08	0.11	0.11	0.17	0.06	0.09	**1.00**									
**12**	0.25	0.29	0.21	0.13	0.16	0.11	0.16	0.18	0.24	0.16	0.14	**1.00**								
**13**	0.27	0.27	0.38	0.36	0.14	0.19	0.28	0.30	0.22	0.17	0.13	0.24	**1.00**							
**14**	0.21	0.23	0.37	0.36	0.12	0.19	0.28	0.28	0.21	0.11	0.17	0.28	***0*.*84***	**1.00**						
**15**	0.18	0.19	0.32	0.25	0.25	0.21	0.28	0.30	0.15	-0.05	0.20	0.36	0.34	0.45	**1.00**					
**16**	0.20	0.23	0.07	0.08	0.15	0.14	0.20	0.22	0.28	0.18	0.02	0.24	0.15	0.16	0.05	**1.00**				
**17**	0.14	0.18	0.15	0.20	0.12	0.12	0.09	0.13	0.04	0.13	0.00	0.09	0.18	0.16	0.11	0.10	**1.00**			
**18**	0.13	0.18	0.11	0.16	0.16	0.10	0.12	0.13	-0.06	0.13	0.05	0.03	0.15	0.11	0.05	0.13	***0*.*59***	**1.00**		
**19**	0.21	0.19	0.26	0.17	0.19	0.18	0.19	0.18	0.15	-0.10	0.09	0.30	0.22	0.19	0.27	0.23	-0.01	-0.07	**1.00**	-0.10–0.46

A goniometer was used in joint mobility measurements and measured to the nearest one degree, except for thumb apposition, palms to floor and patella medial-lateral, which were ordinal variables.

1,2: Shoulder external rotation left and right was assessed by active joint mobility measured in degrees with the participant seated with the upper arms adducted close to the body with elbows flexed to 90 degrees, with the forearm in mid position and thumbs facing up.

3,4: Elbow extension left and right was assessed by passive joint mobility measured in degrees with the participant seated, the arm along the body and forearm in full supination.

5,6: Fifth finger extension left and right was assessed by passive range of motion measured in degrees with the participant seated with the forearm resting on a table with the palm down, the elbow flexed and the adjacent finger stabilized.

7,8: Apposition of the thumb left and right was assessed by passive joint mobility with the participant seated, leaning the elbow on a table and was divided into 4 scores, where 1 = no contact with the forearm, 2 = thumb touches forearm, 3 = thumb digs into forearm easily, 4 = thumb overlaps outside of forearm. Ordinal variable.

9: Palms to floor was assessed with the participant standing through active flexion of the trunk with the knees extended. Palms to floor was divided into 6 scores, where 1 = no contact with the floor, 2 = fingertips touching floor, 3 = fingers touching floor, 4 = palms to floor, 5 = wrists to floor, 6 = forearms to floor. Ordinal variable.

10: Hip abduction was assessed by passive joint mobility measured in degrees with the participant in supine position with straight knees and toes pointing toward the ceiling. No movement was allowed in the pelvis.

11: Hip external rotation was assessed by passive joint mobility measured in degrees with the participant in sitting with the hip to be measured in 90 degrees flexion and neutral in rotation. The knee is flexed 90 degrees with a towel placed under the distal part of the thigh to maintain the horizontal position of the thigh. The leg not to be measured was abducted in the hip joint and the foot is placed on a stool. The subject stabilized with the hands.

12: Hip internal rotation was assessed as the hip external rotation.

13,14: Knee extension left and right was assessed by passive joint mobility measured in degrees with the participant in supine position with knees straight. The heel was placed on a 10 cm high block under with the knee hanging freely in the air.

15: Patella medial-lateral was assessed with the participant in supine with straight knees. Patella was divided into 4 longitudinal quadrants and was passively brought medial and lateral with a total range between 0–8 quadrants. Ordinal variable.

16: Foot flexion was assessed by active joint mobility measured in degrees with the participant half kneeling with hip and knee in 90 degrees, the foot to be measured in front, fully stabilized against the floor.

17,18: Calcaneus valgus test left and right was assessed with the participant standing, groin width distance between feet.

19: Big toe extension was assessed by passive joint mobility measured in degrees with the participant in supine position with ankles and toes in neutral position.

Table 4 shows the result of the Principal component analysis (PCA), to find a subset of joint mobility tests that explains the total variation of all 13 joint mobility tests. The Kaiser-Meyer-Olkin (KMO) measure of sampling adequacy was 0.73. The Kaiser criterion, eigenvalue >1.0, was interpreted to include five components. The PCA was analysed six times, with two to six components. Selecting less or more than five components left a non-interpretable component solution. No cross-loading or freestanding were deleted. Three components had fewer than three items. The five tested components had an explained variance of 9.8% to 14.5% and a cumulative explained variance of maximum 60.3%. Thus, no subset of joint mobility tests describes the variation of all joint mobility tests to define GJH. The Cronbach alpha (α), for the assessment of internal consistency reliability was 0.73.

**Table 4:** Principal component analysis of joint mobility tests among 240 women in early pregnancy.

Variable	Component 1	Component 2	Component 3	Component 4	Component 5
Shoulder external rotation^a^			0.49		
Elbow extension^b^			0.41		
Fifth finger extension^c^				0.69	
Thumb apposition^d^				0.65	
Palms to floor^e^		0.53			
Hip abduction^f^		0.61			
Hip external rotation^g^					0.74
Hip internal rotation^h^	0.42				
Knee extension^i^			0.37		
Patella medial-lateral^j^					0.41
Foot flexion^k^		0.43			
Calcaneus valgus^l^			0.62		
Big toe extension^m^	0.62				
Variance explained (%)^n^	14.5	12.9	12.3	10.8	9.8
Cumulative variance explained (%)	14.5	27.4	39.7	50.6	60.3
Eigenvalue°	3.2	1.4	1.2	1.1	1.0

Principal component analysis with orthogonal varimax rotation showing components loadings above 0.35. A component is calculated using all of the variance of the manifest variables.

A goniometer was used in joint mobility tests measured in degrees. Each joint angle was registered to the nearest 1 degree except for thumb apposition, palms to floor and patella medial-lateral, which were ordinal variables. The result is based on measurement on the non-dominant bodyside.

^a^ Shoulder external rotation was assessed by active joint mobility measured in degrees with the participant seated with the upper arms adducted close to the body with elbows flexed to 90 degrees, with the forearm in mid position and thumbs facing up.

^b^ Elbow extension was assessed by passive joint mobility measured in degrees with the participant seated, the arm along the body and forearm in full supination.

^c^ Fifth finger extension was assessed by passive range of motion measured in degrees with the participant seated with the forearm resting on a table with the palm down, the elbow flexed and the adjacent finger stabilized.

^d^ Apposition of the thumb was assessed by passive joint mobility with the participant seated, leaning the elbow on a table and was divided into 4 scores, where 1 = no contact with the forearm, 2 = thumb touches forearm, 3 = thumb digs into forearm easily, 4 = thumb overlaps outside of forearm. Ordinal variable.

^e^ Palms to floor was assessed with the participant standing through active flexion of the trunk with the knees extended. Palms to floor was divided into 6 scores, where 1 = no contact with the floor, 2 = fingertips touching floor, 3 = fingers touching floor, 4 = palms to floor, 5 = wrists to floor, 6 = forearms to floor. Ordinal variable.

^f^ Hip abduction was assessed by passive joint mobility measured in degrees with the participant in supine position with straight knees and toes pointing toward the ceiling. No movement was allowed in the pelvis.

^g^ Hip external rotation was assessed by passive joint mobility measured in degrees with the participant in sitting with the hip to be measured in 90 degrees flexion and neutral in rotation. The knee is flexed 90 degrees with a towel placed under the distal part of the thigh to maintain the horizontal position of the thigh. The leg not to be measured was abducted in the hip joint and the foot is placed on a stool. The subject stabilized with the hands.

^h^ Hip internal rotation was assessed as the hip external rotation.

^i^ Knee extension was assessed by passive joint mobility measured in degrees with the participant in supine position with knees straight. The heel was placed on a 10 cm high block under with the knee hanging freely in the air.

^j^ Patella medial-lateral was assessed with the participant in supine with straight knees. Patella was divided into 4 longitudinal quadrants and was passively brought medial and lateral with a total range between 0–8 quadrants. Ordinal variable.

^k^ Foot flexion was assessed by active joint mobility measured in degrees with the participant half kneeling with hip and knee in 90 degrees, the foot to be measured in front, fully stabilized against the floor.

^l^ Calcaneus valgus test was assessed with the participant standing, groin width distance between feet.

^m^ Big toe extension was assessed by passive joint mobility measured in degrees with the participant in supine position with ankles and toes in neutral position.

^n^ Variance explained for each component.

° Kaiser criterion, eigenvalues greater than 1.0

Table 5 shows limits to classify joint hypermobility for each of the 13 joint mobility tests on the non-dominant body side, based on the five different SD levels, plus 0.84, plus 1.04, plus 1.28, plus 1.64 and plus 2. The further to the right on the normal distribution, the higher the limit for hypermobility. Plus 2 SD had the lowest prevalence rates.

**Table 5:** Joint hypermobility limits in degrees by different standard deviations among early pregnant women.

Variable	n	+0.84SD (n)	+1.04SD (n)	+1.28SD (n)	+1.64 SD (n)	+2SD (n)
Shoulder external rotation^a^	255	78 (51)	82 (39)	86 (21)	92 (3)	98 (1)
Elbow extension^b^	255	-10 (47)	-11 (37)	-12 (20)	-14 (12)	-16 (4)
Fifth finger extension^c^	255	87 (60)	89 (53)	92 (28)	97 (4)	101 (2)
Thumb apposition^d^	254	2 (37)	2 (37)	2 (37)	3 (1)	3 (1)
Palms to floor^e^	255	3 (72)	3 (72)	3 (72)	4 (18)	4 (18)
Hip abduction^f^	242	41 (43)	42 (35)	44 (19)	46 (13)	49 (4)
Hip external rotation^g^	243	52 (36)	54 (25)	57 (16)	60 (10)	64 (4)
Hip internal rotation^h^	243	59 (48)	61 (36)	64 (25)	67 (18)	71 (7)
Knee extension^i^	255	-7 (52)	-8 (34)	-9 (31)	-11 (18)	-12 (10)
Patella medial-lateral^j^	242	4 (18)	4 (18)	4 (18)	5 (1)	5 (1)
Foot flexion^k^	243	42 (41)	43 (36)	45 (20)	47 (11)	50 (6)
Calcaneus valgus^l^	255	-5 (15)	-5 (15)	-6 (4)	-6 (4)	-7 (2)
Big toe extension^m^	243	109 (58)	112 (31)	115 (19)	119 (13)	124 (2)

A goniometer was used in joint mobility tests and measured to the nearest one degree, except for thumb apposition, palms to floor and patella medial-lateral, which were ordinal variables. The result is based on measurement on the non-dominant bodyside.

^a^ Shoulder external rotation was assessed by active joint mobility measured in degrees with the participant seated with the upper arms adducted close to the body with elbows flexed to 90 degrees, with the forearm in mid position and thumbs facing up.

^b^ Elbow extension was assessed by passive joint mobility measured in degrees with the participant seated, the arm along the body and forearm in full supination.

^c^ Fifth finger extension was assessed by passive range of motion measured in degrees with the participant seated with the forearm resting on a table with the palm down, the elbow flexed and the adjacent finger stabilized.

^d^ Apposition of the thumb was assessed by passive joint mobility with the participant seated, leaning the elbow on a table and was divided into 4 scores, where 1 = no contact with the forearm, 2 = thumb touches forearm, 3 = thumb digs into forearm easily, 4 = thumb overlaps outside of forearm. Ordinal variable.

^e^ Palms to floor was assessed with the participant standing through active flexion of the trunk with the knees extended. Palms to floor was divided into 6 scores, where 1 = no contact with the floor, 2 = fingertips touching floor, 3 = fingers touching floor, 4 = palms to floor, 5 = wrists to floor, 6 = forearms to floor. Ordinal variable.

^f^ Hip abduction was assessed by passive joint mobility measured in degrees with the participant in supine position with straight knees and toes pointing toward the ceiling. No movement was allowed in the pelvis.

^g^ Hip external rotation was assessed by passive joint mobility measured in degrees with the participant in sitting with the hip to be measured in 90 degrees flexion and neutral in rotation. The knee is flexed 90 degrees with a towel placed under the distal part of the thigh to maintain the horizontal position of the thigh. The leg not to be measured was abducted in the hip joint and the foot is placed on a stool. The subject stabilized with the hands.

^h^ Hip internal rotation was assessed as the hip external rotation.

^i^ Knee extension was assessed by passive joint mobility measured in degrees with the participant in supine position with knees straight. The heel was placed on a 10 cm high block under with the knee hanging freely in the air.

^j^ Patella medial-lateral was assessed with the participant in supine with straight knees. Patella was divided into 4 longitudinal quadrants and was passively brought medial and lateral with a total range between 0–8 quadrants. Ordinal variable.

^k^ Foot flexion was assessed by active joint mobility measured in degrees with the participant half kneeling with hip and knee in 90 degrees, the foot to be measured in front, fully stabilized against the floor.

^l^ Calcaneus valgus test was assessed with the participant standing, groin width distance between feet.

^m^ Big toe extension was assessed by passive joint mobility measured in degrees with the participant in supine posit

As a result of no subset of joint mobility tests were found to define GJH, different combinations of major and minor joints in upper and lower limbs and the axial skeleton were systematically developed, S1 Table. The combinations were based on the definition described by Castori et al. [5]. The emphasis was placed on major joints in upper and lower limbs when the definitions were developed. In this study, major joint mobility tests in the upper limb comprised shoulder external rotation, elbow extension and major joint mobility tests in the lower limb comprised knee extension, foot flexion and either of the hip abduction, hip external or internal rotation. Minor joint mobility tests in the upper limb comprised the fifth finger extension and thumb apposition and minor joint mobility tests in the lower limb comprised patella medial-lateral, calcaneus valgus and big toe extension. The axial skeleton comprised the palms to floor test. The combinations were evaluated for each of the five different SD levels. If there were missing in any of the joint mobility tests in major upper or lower and minor upper or lower, these were classified as missing, to avoid misclassification.

Table 6 presents the distribution of eight combinations of major and minor joints, in upper and lower limbs and the axial skeleton to define GJH, divided into the five different SD levels. The prevalence of GJH by the eight definitions were between 0.0 and 13.8%. The first three combinations and the uppermost SD levels, plus 2 and plus 1.64 entailed the lowest prevalences. Combination seven to eight and SD levels plus 1.04 and 0.84 entailed the highest prevalences. Any of the eight combinations were fulfilled by 38 (15.8%) women at SD level 0.84, 22 (9.2%) women at SD level 1.04, 11 (4.6%) women at SD level 1.28, one woman (0.4%) at SD level 1.64 and none at SD level 2. The eight combinations showed a various distribution of hypermobile joint mobility tests between the women. The distribution of hypermobile joint mobility tests in the eight combinations is compiled in S2 Table.

**Table 6:** Number of women with generalized joint hypermobility by five joint hypermobility limits and eight combinations.

Combination	N	+0.84 SD n (%)	+1.04 SD n (%)	+1.28 SD n (%)	+1.64 SD n (%)	+2 SD n (%)
1^a^	240	10 (4.2)	6 (2.5)	2 (0.8)	0 (0.0)	0 (0.0)
2^b^	240	9 (3.8)	6 (2.5)	3 (1.2)	0 (0.0)	0 (0.0)
3^c^	240	9 (3.8)	7 (2.9)	2 (0.8)	0 (0.0)	0 (0.0)
4^d^	240	13 (5.4)	10 (4.2)	5 (2.1)	0 (0.0)	0 (0.0)
5^e^	240	18 (7.5)	10 (4.2)	3 (1.2)	0 (0.0)	0 (0.0)
6^f^	240	18 (7.5)	9 (3.8)	3 (1.2)	0 (0.0)	0 (0.0)
7^g^	240	18 (7.5)	13 (5.4)	6 (2.5)	0 (0.0)	0 (0.0)
8^h^	240	33 (13.8)	19 (7.9)	10 (4.2)	1 (0.4)	0 (0.0)
Any of the eight combination		38 (15.8)	22 (9.2)	11 (4.6)	1 (0.4)	0 (0.0)

^a^ Combination 1: joint hypermobility in at least one major joint in upper limb, in at least one major joint in lower limb, in at least one minor joint in upper limb and in at least one minor joint in lower limb and in the axial skeleton.

^b^ Combination 2: joint hypermobility in at least three major joints distributed between upper and lower limbs and joint hypermobility in at least one minor joint in lower limb and in the axial skeleton.

^c^ Combination 3: joint hypermobility in at least three major joints distributed between upper and lower limbs and joint hypermobility in at least one minor joint in upper limb and in the axial skeleton.

^d^ Combination 4: joint hypermobility in at least three major joints distributed between upper and lower limbs and the axial skeleton.

^e^ Combination 5: joint hypermobility in at least one major joint in upper limb, in at least one major joint in lower limb, in at least one minor joint in upper limb and in at least one minor joint in lower limb.

^f^ Combination 6: joint hypermobility in at least three major joints distributed between upper and lower limbs and joint hypermobility in at least one minor joint in lower limb.

^g^ Combination 7: joint hypermobility in at least three major joints distributed between upper and lower limbs and joint hypermobility in at least one minor joint in upper limb.

^h^ Combination 8: joint hypermobility in at least three major joints distributed between upper and lower limbs.

## Discussion

No subset of the included unilateral joint mobility tests could explain the total variation of all joint mobility tests. However, with use of standardized joint hypermobility limits and different combinations of major and minor joints in upper and lower limbs and axial skeleton an array of definitions of generalized joint hypermobility is presented. The different definitions showed a wide prevalence of generalized joint hypermobility and there was a clear variation in how the hypermobile joint mobility tests were distributed between the women.

Anatomical differences such as joint type and articular soft tissue structure as well as the statistical analysis may explain why a subset of joint mobility tests to define GJH was not found. Since both the correlation analysis and the PCA were pointing in the same direction, this suggests that the statistical analysis would not account for the strongest explanation. The weak correlation result between the joint mobility tests, without taking into account the bilateral joint mobility tests, was in line with two other studies [17, 25]. The study by Silman et al. also found that joint mobility at one site, could not predict joint mobility at the other sites measured [25]. Finding no relationship between the different joint mobility tests was sursprising. Different joint types and various construction of articular soft tissue structure seem to be important and need to be included in the assessment of GJH.

To the best of our knowledge, there are no previous studies to classify limits of joint hypermobility for each separate joint mobility test, in a systematic way. To classify joint hypermobility, in each joint mobility test, the current study presents five different SD levels. To avoid classification bias Fairbank et al. [17] suggest the same deviation from the mean should be used for all joint mobility tests. Both the BeS and the HdM uses arbitrary limits to classify joint hypermobility within the score, which introduces bias in the classification of GJH. By using the same SD level, uniform limits are provided for all included joint mobility tests in the definition of GJH. No joint is therefore given more weight than any other. As in the study by Silman et al. [25] a very small number of women were at the top of the distribution of joint mobility.

In the present study, GJH appears to be distributed in different combinations of joint mobility tests, which was also seen in the study by Larsson et al. [26]. Additionally, by using combinations of joint mobility tests in major and minor joints in upper and lower limbs and the axial skeleton, the definition of GJH is facilitated and not limited to selected joints. Any or some of the eight definitions may well be used to define GJH as a criterion in heritable connective tissue disorders as well as to evaluate various pain conditions. While using a cut-off level to define GJH, as usually used in GJH scores, there is always a risk that the definition of GJH can be limited to end up in one half of the body. However, including several joint mobility tests could mean the assessment becomes more time-consuming.

Selecting joints, to define GJH, in this study the non-dominant side of the body was chosen to reduce the bias of high correlation of bilaterally measured joint mobility and to reduce the negative impact on joint mobility due to a more frequent musculature on the dominant half of the body. Also, we included joints to represent a variety of anatomical differences such as, joint type and articular soft tissue structure, as well as a combination of major- and minor joints in both upper and lower limbs and the axial skeleton. In most previous studies the selected joints to define GJH are not described. An exception is the HdM criteria [11] which was based on the most prevalent and reliable joint mobility tests on the non-dominant bodyside.

To define GJH, Grahame proposed as early as 1999 that “other areas worth looking into are proximal and distal interphalangeal joints, shoulders, cervical spine, hips, patellae, ankles, hind and forefeet, as well as metacarpophalangeal joints”, otherwise, joint hypermobility may pass undetected if only few joints are included [27]. This is in line with our study where the conclusion is that several joint mobility tests are needed, as no subset for the definition of GJH was found.

In the present study, angular joint mobility was used. In the two scores, The Upper limb assessment score and Lower Limb Assessment Score [12, 13] tests for joint instability and translation are also included. The result in our study supports the use of both scores if used together. However, clinical tests of joint instability and translation are difficult to evaluate in a standardized way in the clinic.

A limitation, in the present study, is that we cannot exclude that there are further joint mobility tests or directions of movements, which we did not include, that could result in a subset of joint mobility tests to define GJH. Another limitation of the study was not including specific joint mobility test for the axial skeleton, especially the neck which is clinically important. A further limitation could be that the study population were not representative for the assessment of GJH which could affect the prevalence of GJH.

A major strength of the study, was that the joint mobility tests followed a thoroughly structured protocol [20]. This increases the possibility of reliable and comparable measurements. Another strength was the inclusion of joint mobility tests in anatomical different joint types, articular soft tissue structure, major and minor joints in upper and lower limbs and the axial skeleton. This facilitates the definition of GJH and includes common problem areas relevant for GJH [28–31]. Furthermore, by including a homogeneous group, women with similar origin, age and hormonal status, the definition of GJH, for the studied population, is facilitated. Yet, the result is only comparable with a similar studygroup.

## Conclusion

It is probably not possible to choose a subset of joint mobility tests to define GJH. In order not to overlook GJH, a broader investigation of different joint types and sizes of joints appears to be needed. The prevalence of GJH is dependent on which joint hypermobility limit and combination of major and minor joints in upper and lower limbs and the axial skeleton is used. Also, there was a clear variation in how the hypermobile joint mobility tests were distributed between the women.

## Supporting information

S1 Table Combinations to define generalized joint hypermobility. (DOCX)

S2 Table The distribution of hypermobile joint mobility tests in the eight combinations to define generalized joint hypermobility. (DOCX)
