# Supplementary material for: “Could a subset of joint mobility tests define generalized joint hypermobility?”: A descriptive observational inception study
Source: PLoS One. 2024 Apr 18;19(4):e0298649. doi: 10.1371/journal.pone.0298649 (PMC11025819; doi:10.1371/journal.pone.0298649)
Supplement: S1 Table — (DOCX) [file pone.0298649.s001.docx]

| Combinations | Major upper^a^ | Minor upper^b^ | Major lower^c^ | Minor lower^d^ | Axial skeleton^e^ |
| --- | --- | --- | --- | --- | --- |
| Combination 1^f^ | ≥1 | ≥1 | ≥1 | ≥1 | ≥1 |
| Combination 2^g^ | ≥ 1 or ≥ 2 |  | ≥ 2 or ≥ 1 | ≥1 | ≥1 |
| Combination 3^h^ | ≥ 1 or ≥ 2 | ≥1 | ≥ 2 or ≥ 1 |  | ≥1 |
| Combination 4^i^ | ≥ 1 or ≥ 2 |  | ≥ 2 or ≥ 1 |  | ≥1 |
| Combination 5^j^ | ≥1 | ≥1 | ≥1 | ≥1 |  |
| Combination 6^k^ | ≥ 1 or ≥ 2 |  | ≥ 2 or ≥ 1 | ≥1 |  |
| Combination 7^l^ | ≥ 1 or ≥ 2 | ≥1 | ≥ 2 or ≥ 1 |  |  |
| Combination 8^m^ | ≥ 1 or ≥ 2 |  | ≥ 2 or ≥ 1 |  |  |

^a^ Shoulder external rotation and elbow extension.

^b^ Fifth finger extension and thumb apposition.

^c^ Knee extension, foot flexion and either of the hip abduction, hip external or internal rotation.

^d^ Patella medial-lateral, calcaneus valgus and big toe extension.

^e^ The axial skeleton comprised by the palms to floor test.

^f^ Combination 1: joint hypermobility in at least one major joint in upper limb, in at least one major joint in lower limb, in at least one minor joint in upper limb and in at least one minor joint in lower limb and in the axial skeleton.

^g^ Combination 2: joint hypermobility in at least three major joints distributed between upper and lower limbs and joint hypermobility in at least one minor joint in lower limb and in the axial skeleton.

^h^ Combination 3: joint hypermobility in at least three major joints distributed between upper and lower limbs and joint hypermobility in at least one minor joint in upper limb and in the axial skeleton.

^i^ Combination 4: joint hypermobility in at least three major joints distributed between upper and lower limbs and the axial skeleton.

^j^ Combination 5: joint hypermobility in at least one major joint in upper limb, in at least one major joint in lower limb, in at least one minor joint in upper limb and in at least one minor joint in lower limb.

^k^ Combination 6: joint hypermobility in at least three major joints distributed between upper and lower limbs and joint hypermobility in at least one minor joint in lower limb.

^l^ Combination 7: joint hypermobility in at least three major joints distributed between upper and lower limbs and joint hypermobility in at least one minor joint in upper limb.
^m^ Combination 8: joint hypermobility in at least three major joints distributed between upper and lower limbs.

S1 Table Combinations to define generalized joint hypermobility
