# Supplementary material for: “Could a subset of joint mobility tests define generalized joint hypermobility?”: A descriptive observational inception study
Source: PLoS One. 2024 Apr 18;19(4):e0298649. doi: 10.1371/journal.pone.0298649 (PMC11025819; doi:10.1371/journal.pone.0298649)
Supplement: S2 Table — (DOCX) [file pone.0298649.s002.docx]

**Plus 2 SD**

Combination 1, **2.0 SD**, 0 women

| Major upper | Minor upper | Major lower | Minor lower | Axial skeleton |
| --- | --- | --- | --- | --- |

Combination 2, **2.0 SD**, 0 women

| Major upper |  | Major lower | Minor lower | Axial skeleton |
| --- | --- | --- | --- | --- |

Combination 3, **2.0 SD**, 0 women

| Major upper | Minor upper | Major lower |  | Axial skeleton |
| --- | --- | --- | --- | --- |

Combination 4, **2.0 SD**, 0 women

| Major upper |  | Major lower |  | Axial skeleton |
| --- | --- | --- | --- | --- |

Combination 5, **2.0 SD**, 0 women

| Major upper | Minor upper | Major lower | Minor lower |  |
| --- | --- | --- | --- | --- |

Combination 6, **2.0 SD**, 0 women

| Major upper |  | Major lower | Minor lower |  |
| --- | --- | --- | --- | --- |

Combination 7, **2.0 SD**, 0 women

| Major upper | Minor upper | Major lower |  |  |
| --- | --- | --- | --- | --- |

Combination 8, **2.0 SD**, 0 women

| Major upper |  | Major lower |  |  |
| --- | --- | --- | --- | --- |

**Plus 1.64 SD**

Combination 1, **1.64 SD**, 0 women

| Major upper | Minor upper | Major lower | Minor lower | Axial skeleton |
| --- | --- | --- | --- | --- |

Combination 2, **1.64 SD**, 0 women

| Major upper |  | Major lower | Minor lower | Axial skeleton |
| --- | --- | --- | --- | --- |

Combination 3, **1.64 SD**, 0 women

| Major upper | Minor upper | Major lower |  | Axial skeleton |
| --- | --- | --- | --- | --- |

Combination 4, **1.64 SD**, 0 women

| Major upper |  | Major lower |  | Axial skeleton |
| --- | --- | --- | --- | --- |

Combination 5, **1.64 SD**, 0 women

| Major upper | Minor upper | Major lower | Minor lower |  |
| --- | --- | --- | --- | --- |

Combination 6, **1.64 SD**, 0 women

| Major upper |  | Major lower | Minor lower |  |
| --- | --- | --- | --- | --- |

Combination 7, **1.64 SD**, 0 women

| Major upper | Minor upper | Major lower |  |  |
| --- | --- | --- | --- | --- |

Combination 8, **1.64 SD**, 1 women

| Major upper |  | Major lower |  |  |
| --- | --- | --- | --- | --- |
| 2 |  | 2 |  |  |

**Plus 1.28 SD**

Combination 1, **1.28 SD**, 2 women

| Major upper | Minor upper | Major lower | Minor lower | Axial skeleton |
| --- | --- | --- | --- | --- |
| 1 | 1 | 2 | 1 | 1 |
| 2 | 1 | 2 | 1 | 1 |

Combination 2, **1.28 SD**, 3 women

| Major upper |  | Major lower | Minor lower | Axial skeleton |
| --- | --- | --- | --- | --- |
| 1 |  | 2 | 1 | 1 |
| 1 |  | 2 | 1 | 1 |
| 2 |  | 2 | 1 | 1 |

Combination 3, **1.28 SD**, 2 women

| Major upper | Minor upper | Major lower |  | Axial skeleton |
| --- | --- | --- | --- | --- |
| 1 | 1 | 2 |  | 1 |
| 2 | 1 | 2 |  | 1 |

Combination 4, **1.28 SD**, 5 women

| Major upper |  | Major lower |  | Axial skeleton |
| --- | --- | --- | --- | --- |
| 1 |  | 2 |  | 1 |
| 1 |  | 2 |  | 1 |
| 1 |  | 2 |  | 1 |
| 1 |  | 2 |  | 1 |
| 2 |  | 2 |  | 1 |

Combination 5, **1.28 SD**, 3 women

| Major upper | Minor upper | Major lower | Minor lower |  |
| --- | --- | --- | --- | --- |
| 1 | 1 | 2 | 1 |  |
| 2 | 1 | 2 | 1 |  |
| 1 | 1 | 1 | 1 |  |

Combination 6, **1.28 SD**, 3 women

| Major upper |  | Major lower | Minor lower |  |
| --- | --- | --- | --- | --- |
| 1 |  | 2 | 1 |  |
| 1 |  | 2 | 1 |  |
| 2 |  | 2 | 1 |  |

Combination 7, **1.28 SD**, 6 women

| Major upper | Minor upper | Major lower |  |  |
| --- | --- | --- | --- | --- |
| 1 | 1 | 2 |  |  |
| 1 | 1 | 2 |  |  |
| 2 | 1 | 1 |  |  |
| 1 | 1 | 3 |  |  |
| 2 | 1 | 2 |  |  |
| 2 | 1 | 2 |  |  |

Combination 8, **1.28 SD**, 10 women

| Major upper |  | Major lower |  |  |
| --- | --- | --- | --- | --- |
| 1 |  | 2 |  |  |
| 1 |  | 2 |  |  |
| 1 |  | 2 |  |  |
| 1 |  | 2 |  |  |
| 2 |  | 1 |  |  |
| 1 |  | 3 |  |  |
| 1 |  | 2 |  |  |
| 1 |  | 2 |  |  |
| 2 |  | 2 |  |  |
| 2 |  | 2 |  |  |

**Plus 1.04 SD**

Combination 1, **1.04 SD**, 6 women

| Major upper | Minor upper | Major lower | Minor lower | Axial skeleton |
| --- | --- | --- | --- | --- |
| 2 | 1 | 2 | 1 | 1 |
| 1 | 2 | 2 | 1 | 1 |
| 2 | 2 | 2 | 1 | 1 |
| 1 | 1 | 1 | 1 | 1 |
| 2 | 2 | 1 | 1 | 1 |
| 1 | 2 | 2 | 1 | 1 |

Combination 2, **1.04 SD**, 6 women

| Major upper |  | Major lower | Minor lower | Axial skeleton |
| --- | --- | --- | --- | --- |
| 2 |  | 2 | 1 | 1 |
| 1 |  | 2 | 1 | 1 |
| 1 |  | 2 | 1 | 1 |
| 2 |  | 2 | 1 | 1 |
| 2 |  | 1 | 1 | 1 |
| 1 |  | 2 | 1 | 1 |

Combination 3, **1.04 SD**, 7 women

| Major upper | Minor upper | Major lower |  | Axial skeleton |
| --- | --- | --- | --- | --- |
| 2 | 1 | 2 |  | 1 |
| 2 | 1 | 2 |  | 1 |
| 1 | 1 | 2 |  | 1 |
| 1 | 2 | 2 |  | 1 |
| 2 | 2 | 2 |  | 1 |
| 2 | 2 | 1 |  | 1 |
| 1 | 2 | 2 |  | 1 |

Combination 4, **1.04 SD**, 10 women

| Major upper |  | Major lower |  | Axial skeleton |
| --- | --- | --- | --- | --- |
| 2 |  | 2 |  | 1 |
| 2 |  | 2 |  | 1 |
| 1 |  | 2 |  | 1 |
| 1 |  | 2 |  | 1 |
| 1 |  | 2 |  | 1 |
| 1 |  | 2 |  | 1 |
| 2 |  | 2 |  | 1 |
| 1 |  | 2 |  | 1 |
| 2 |  | 1 |  | 1 |
| 1 |  | 2 |  | 1 |

Combination 5, **1.04 SD**, 10 women

| Major upper | Minor upper | Major lower | Minor lower |  |
| --- | --- | --- | --- | --- |
| 2 | 1 | 2 | 1 |  |
| 1 | 1 | 1 | 1 |  |
| 1 | 2 | 2 | 1 |  |
| 2 | 2 | 2 | 1 |  |
| 1 | 1 | 1 | 1 |  |
| 2 | 2 | 1 | 1 |  |
| 1 | 1 | 1 | 1 |  |
| 1 | 2 | 2 | 1 |  |
| 1 | 2 | 2 | 1 |  |
| 1 | 1 | 2 | 1 |  |

Combination 6, **1.04 SD**, 9 women

| Major upper |  | Major lower | Minor lower |  |
| --- | --- | --- | --- | --- |
| 2 |  | 2 | 1 |  |
| 1 |  | 2 | 1 |  |
| 1 |  | 2 | 1 |  |
| 2 |  | 2 | 1 |  |
| 2 |  | 1 | 1 |  |
| 1 |  | 2 | 1 |  |
| 1 |  | 2 | 1 |  |
| 1 |  | 2 | 1 |  |
| 2 |  | 1 | 1 |  |

Combination 7, **1.04 SD**, 13 women

| Major upper | Minor upper | Major lower |  |  |
| --- | --- | --- | --- | --- |
| 2 | 1 | 2 |  |  |
| 2 | 1 | 2 |  |  |
| 1 | 1 | 2 |  |  |
| 1 | 1 | 2 |  |  |
| 2 | 1 | 1 |  |  |
| 1 | 1 | 3 |  |  |
| 1 | 2 | 2 |  |  |
| 2 | 2 | 2 |  |  |
| 2 | 2 | 1 |  |  |
| 1 | 2 | 2 |  |  |
| 1 | 2 | 2 |  |  |
| 2 | 1 | 2 |  |  |
| 1 | 1 | 2 |  |  |

Combination 8, **1.04 SD**, 19 women

| Major upper |  | Major lower |  |  |
| --- | --- | --- | --- | --- |
| 1 |  | 2 |  |  |
| 2 |  | 2 |  |  |
| 2 |  | 2 |  |  |
| 1 |  | 2 |  |  |
| 1 |  | 2 |  |  |
| 2 |  | 1 |  |  |
| 1 |  | 3 |  |  |
| 1 |  | 2 |  |  |
| 1 |  | 2 |  |  |
| 1 |  | 2 |  |  |
| 2 |  | 2 |  |  |
| 1 |  | 2 |  |  |
| 2 |  | 1 |  |  |
| 1 |  | 2 |  |  |
| 1 |  | 2 |  |  |
| 2 |  | 1 |  |  |
| 2 |  | 2 |  |  |
| 1 |  | 2 |  |  |
| 2 |  | 1 |  |  |

**Plus 0.84 SD**

Combination 1, **0.84 SD**, 10 women

| Major upper | Minor upper | Major lower | Minor lower | Axial skeleton |
| --- | --- | --- | --- | --- |
| 1 | 2 | 1 | 1 | 1 |
| 2 | 1 | 2 | 1 | 1 |
| 1 | 2 | 3 | 1 | 1 |
| 1 | 2 | 3 | 1 | 1 |
| 1 | 2 | 2 | 1 | 1 |
| 2 | 2 | 2 | 1 | 1 |
| 1 | 1 | 1 | 2 | 1 |
| 1 | 1 | 1 | 1 | 1 |
| 2 | 2 | 2 | 1 | 1 |
| 1 | 2 | 2 | 1 | 1 |

Combination 2, **0.84 SD**, 9 women

| Major upper |  | Major lower | Minor lower | Axial skeleton |
| --- | --- | --- | --- | --- |
| 1 |  | 2 | 1 | 1 |
| 2 |  | 2 | 1 | 1 |
| 1 |  | 3 | 1 | 1 |
| 1 |  | 3 | 1 | 1 |
| 1 |  | 2 | 1 | 1 |
| 1 |  | 2 | 2 | 1 |
| 2 |  | 2 | 1 | 1 |
| 2 |  | 2 | 1 | 1 |
| 1 |  | 2 | 1 | 1 |

Combination 3, **0.84 SD**, 9 women

| Major upper | Minor upper | Major lower |  | Axial skeleton |
| --- | --- | --- | --- | --- |
| 2 | 1 | 2 |  | 1 |
| 2 | 1 | 2 |  | 1 |
| 1 | 1 | 2 |  | 1 |
| 1 | 2 | 3 |  | 1 |
| 1 | 2 | 3 |  | 1 |
| 1 | 2 | 2 |  | 1 |
| 2 | 2 | 2 |  | 1 |
| 2 | 2 | 2 |  | 1 |
| 1 | 2 | 2 |  | 1 |

Combination 4, **0.84 SD**, 13 women

| Major upper |  | Major lower |  | Axial skeleton |
| --- | --- | --- | --- | --- |
| 1 |  | 2 |  | 1 |
| 2 |  | 2 |  | 1 |
| 2 |  | 2 |  | 1 |
| 1 |  | 2 |  | 1 |
| 1 |  | 3 |  | 1 |
| 1 |  | 3 |  | 1 |
| 1 |  | 2 |  | 1 |
| 2 |  | 2 |  | 1 |
| 1 |  | 2 |  | 1 |
| 2 |  | 2 |  | 1 |
| 1 |  | 2 |  | 1 |
| 2 |  | 2 |  | 1 |
| 1 |  | 2 |  | 1 |

Combination 5, **0.84 SD**, 18 women

| Major upper | Minor upper | Major lower | Minor lower |  |
| --- | --- | --- | --- | --- |
| 1 | 2 | 1 | 1 |  |
| 2 | 1 | 2 | 1 |  |
| 1 | 2 | 3 | 1 |  |
| 1 | 2 | 3 | 1 |  |
| 1 | 1 | 1 | 2 |  |
| 1 | 2 | 2 | 1 |  |
| 1 | 1 | 1 | 1 |  |
| 2 | 2 | 2 | 1 |  |
| 1 | 1 | 1 | 2 |  |
| 1 | 1 | 1 | 1 |  |
| 2 | 2 | 2 | 1 |  |
| 2 | 1 | 1 | 2 |  |
| 1 | 2 | 2 | 1 |  |
| 1 | 2 | 2 | 1 |  |
| 1 | 1 | 2 | 1 |  |
| 2 | 2 | 2 | 1 |  |
| 1 | 1 | 2 | 2 |  |
| 2 | 1 | 2 | 1 |  |

Combination 6, **0.84 SD**, 18 women

| Major upper |  | Major lower | Minor lower |  |
| --- | --- | --- | --- | --- |
| 1 |  | 2 | 1 |  |
| 2 |  | 2 | 1 |  |
| 2 |  | 1 | 1 |  |
| 2 |  | 2 | 1 |  |
| 1 |  | 3 | 1 |  |
| 1 |  | 3 | 1 |  |
| 1 |  | 2 | 1 |  |
| 1 |  | 2 | 1 |  |
| 1 |  | 2 | 2 |  |
| 2 |  | 2 | 1 |  |
| 2 |  | 2 | 1 |  |
| 2 |  | 1 | 2 |  |
| 1 |  | 2 | 1 |  |
| 1 |  | 2 | 1 |  |
| 1 |  | 2 | 1 |  |
| 2 |  | 2 | 1 |  |
| 1 |  | 2 | 2 |  |
| 2 |  | 2 | 1 |  |

Combination 7, **0.84 SD**, 18 women

| Major upper | Minor upper | Major lower |  |  |
| --- | --- | --- | --- | --- |
| 2 | 1 | 2 |  |  |
| 2 | 1 | 2 |  |  |
| 1 | 1 | 2 |  |  |
| 1 | 1 | 2 |  |  |
| 1 | 2 | 3 |  |  |
| 1 | 2 | 3 |  |  |
| 2 | 1 | 1 |  |  |
| 1 | 1 | 3 |  |  |
| 1 | 2 | 2 |  |  |
| 2 | 2 | 2 |  |  |
| 2 | 2 | 2 |  |  |
| 2 | 1 | 1 |  |  |
| 1 | 2 | 2 |  |  |
| 1 | 2 | 2 |  |  |
| 1 | 1 | 2 |  |  |
| 2 | 2 | 2 |  |  |
| 1 | 1 | 2 |  |  |
| 2 | 1 | 2 |  |  |

Combination 8, **0.84 SD**, 33 women

| Major upper |  | Major lower |  |  |
| --- | --- | --- | --- | --- |
| 1 |  | 2 |  |  |
| 1 |  | 3 |  |  |
| 1 |  | 2 |  |  |
| 2 |  | 2 |  |  |
| 2 |  | 1 |  |  |
| 2 |  | 2 |  |  |
| 2 |  | 1 |  |  |
| 2 |  | 2 |  |  |
| 1 |  | 2 |  |  |
| 1 |  | 2 |  |  |
| 1 |  | 2 |  |  |
| 1 |  | 3 |  |  |
| 1 |  | 3 |  |  |
| 2 |  | 1 |  |  |
| 1 |  | 3 |  |  |
| 1 |  | 2 |  |  |
| 1 |  | 2 |  |  |
| 2 |  | 2 |  |  |
| 1 |  | 2 |  |  |
| 2 |  | 2 |  |  |
| 1 |  | 2 |  |  |
| 1 |  | 2 |  |  |
| 2 |  | 2 |  |  |
| 1 |  | 2 |  |  |
| 2 |  | 1 |  |  |
| 1 |  | 2 |  |  |
| 1 |  | 2 |  |  |
| 1 |  | 2 |  |  |
| 2 |  | 2 |  |  |
| 1 |  | 2 |  |  |
| 2 |  | 2 |  |  |
| 1 |  | 2 |  |  |
| 2 |  | 2 |  |  |

S2 Table The distribution of hypermobile joint mobility tests in the eight combinations to define generalized joint hypermobility
